# Supplementary material for: Integrated Biomarker Analysis Reveals L1CAM as a Potential Stratification Marker for No Specific Molecular Profile High-Risk Endometrial Carcinoma
Source: Cancers (Basel). 2022 Nov 3;14(21):5429. doi: 10.3390/cancers14215429 (PMC9658459; doi:10.3390/cancers14215429)
Supplement: Supplementary file 1 [file cancers-14-05429-s001.zip › cancers-1817203-supplementary.pdf]

SUPPLEMENTARY MATERIALS

Table S1. Amplicons in the custom-made high-risk endometrial carcinoma NGS panel. For each amplicon the start and end position are presented in reference genome hg 19. Median amplicon length was 93 base pairs (range 77-104).

Chromosome	Position start	Position end	Amplicon name	Pool #	Gene	Exon
3	41265956	41266042	AMPL7170083667	1	CTNNB1	3
3	41266034	41266122	AMPL7170083217	2	CTNNB1	3
3	41266111	41266202	AMPL7170080333	1	CTNNB1	3
3	41266186	41266273	AMPL7170084011	2	CTNNB1	3
12	133202644	133202733	AMPL7170315715	1	POLE	46
12	133202720	133202820	AMPL7170315322	2	POLE	46
12	133202783	133202873	AMPL7170087761	1	POLE	46
12	133202871	133202968	AMPL7170313847	2	POLE	46
12	133212433	133212524	AMPL7170315610	1	POLE	42
12	133212495	133212585	AMPL7170311719	2	POLE	42
12	133212566	133212650	AMPL7170312360	1	POLE	42
12	133218307	133218399	AMPL7170315668	2	POLE	39
12	133218354	133218445	AMPL7170087850	1	POLE	39
12	133218433	133218533	AMPL7170311573	2	POLE	39
12	133219086	133219183	AMPL7170604674	2	POLE	37
12	133219148	133219237	AMPL7170604666	1	POLE	37
12	133219234	133219330	AMPL7170604675	2	POLE	37
12	133219372	133219455	AMPL7170315086	1	POLE	36
12	133219435	133219539	AMPL7170314571	2	POLE	36
12	133219489	133219589	AMPL7170087809	1	POLE	36
12	133219584	133219679	AMPL7170604772	2	POLE	36
12	133226247	133226343	AMPL7170604781	1	POLE	30
12	133226326	133226430	AMPL7170604778	2	POLE	30
12	133226407	133226497	AMPL7170604789	1	POLE	30
12	133235865	133235953	AMPL7170604771	2	POLE	26
12	133235936	133236033	AMPL7170312021	1	POLE	26
12	133236024	133236110	AMPL7170087651	2	POLE	26
12	133244045	133244139	AMPL7170087913	1	POLE	20
12	133244122	133244218	AMPL7170313927	2	POLE	20
12	133244214	133244304	AMPL7170312937	1	POLE	20
12	133244900	133244985	AMPL7170086397	2	POLE	19
12	133244973	133245066	AMPL7170311520	1	POLE	19
12	133245062	133245165	AMPL7170313170	2	POLE	19
12	133249699	133249794	AMPL7170102163	1	POLE	14
12	133249790	133249882	AMPL7170102124	2	POLE	14
12	133250088	133250187	AMPL7170092315	1	POLE	13
12	133250222	133250313	AMPL7170084021	1	POLE	13
12	133251936	133252033	AMPL7170083158	2	POLE	12

Chromosome	Position start	Position end	Amplicon name	Pool #	Gene	Exon
12	133252301	133252394	AMPL7170084401	1	POLE	11
12	133252349	133252436	AMPL7170083946	2	POLE	11
12	133252660	133252753	AMPL7170083794	1	POLE	10
12	133252707	133252797	AMPL7170083816	2	POLE	10
12	133252794	133252875	AMPL7170577368	1	POLE	10
12	133253118	133253211	AMPL7170083836	1	POLE	9
12	133253176	133253253	AMPL7170577365	2	POLE	9

Table S2. Quality control of next-generation sequencing output files for each patient in terms of total coverage, on-target sequencing, average depth and uniformity.

Patient ID	Coverage (bp)	On target (%)	Mean depth (x)	Uniformity (%)
EC-BS01	717505	88.94	16197	97.6
EC-BS02	123871	84.99	2671	99.4
EC-BS03	1044977	76.25	19800	96.09
EC-BS05	671181	92.02	15801	98.16
EC-BS06	138709	89.74	3154	97.33
EC-BS07	161315	90.14	3707	97,33
EC-BS08	140203	86.17	3039	97.33
EC-BS09	93492	71.56	1616	99.4
EC-BS10	141754	85.28	3057	95.97
EC-BS11	139934	85.9	3056	97.33
EC-BS12	165279	80.79	3360	97.33
EC-BS13	184406	83.61	3906	97.33
EC-BS14	62406	61.8	902	97.33
EC-BS15	157216	82.69	3274	97.33
EC-BS16	153916	89.16	3476	97.33
EC-BS17	203797	87.98	4467	97.33
EC-BS18	458435	88.66	10206	97.33
EC-BS19	139236	87.03	3005	99.38
EC-BS20	274626	74.79	4992	97.29
EC-BS21	95431	56.75	1170	97.15
EC-BS22	116644	90.03	2703	96.99
EC-BS23	113989	72.3	1994	96.73
EC-BS24	125215	86.22	2751	96.99
EC-BS25	89312	69.9	1531	96.57
EC-BS26	150676	79.13	2994	97.33
EC-BS27	88674	78.18	1715	97.09
EC-BS28	148243	74.64	2783	96.65
EC-BS29	84024	76.46	1636	95.31
EC-BS30	74570	88.65	1666	96.05
EC-BS33	169424	90.51	3888	96.72
EC-BS34	101985	80.94	2040	99.36
EC-BS35	113388	80.52	2309	94.94

Patient ID	Coverage (bp)	On target (%)	Mean depth (x)	Uniformity (%)
EC-BS37	216196	88.88	4859	97.32
EC-BS38	587651	73.41	10839	97.33
EC-BS39	91557	88.06	2032	96.62
EC-BS40	209573	84.09	4475	97.11
EC-BS41	234259	92.39	5464	96.01
EC-BS42	114937	84.28	2448	97.21
EC-BS43	64992	85.06	1401	95.93
EC-BS44	120576	91.41	2816	97.25
EC-BS46	68537	90.53	1597	96.31
EC-BS48	51673	89.93	1194	96.99
EC-BS49	110093	89.05	2485	97.16
EC-BS50	563138	77.14	10866	97.33
EC-BS51	62384	84.05	1317	97.43
EC-BS52	558023	87.78	12531	97.13
EC-BS53	497697	88.69	11232	96.24
EC-BS54	88381	91.35	2066	96.24
EC-BS55	703517	81.53	14256	96.78
EC-BS56	173878	82.73	3623	97.41
EC-BS57	198888	90.07	4656	96.09
EC-BS58	121769	87.92	2728	96.51
EC-BS59	87772	87.9	1943	96.15
EC-BS60	93819	89.77	2138	97.33
EC-BS61	126349	89.99	2902	97.33
EC-BS62	64812	84.58	1386	97.25
EC-BS63	44197	84.27	914	94.71
EC-BS64	295241	90.96	6883	96.24
EC-BS65	36608	92.53	864	97.12
EC-BS66	203599	88.15	4561	97.15
EC-BS67	31737	89.37	713	95.71
EC-BS68	35488	88.5	787	97.33
EC-BS69	25820	87.13	568	97.17
EC-BS70	143108	86.74	3140	97.33
EC-BS71	165707	92.16	3833	95.94
EC-BS72	371935	83.49	7809	97.07
EC-BS73	144491	95.16	3512	93.24
EC-BS74	157920	85.29	3394	97.33
EC-BS75	243404	95.03	5934	92.86
EC-BS76	586620	89.24	13308	95.98
EC-BS77	421039	92.72	9821	96.24
EC-BS78	426066	90.39	9710	94.67
EC-BS79	143124	86.53	3123	97.33
EC-BS80	147611	95.92	3576	94.92
EC-BS81	388286	93.9	8988	94.48
EC-BS83	539496	82.86	11201	98.37
EC-BS84	107795	81.61	2215	96.8
EC-BS86	159732	90.29	3592	96.47
EC-BS87	708899	47.22	7892	94
EC-BS88	193859	84.08	4098	96.46
EC-BS89	749355	77.87	14288	96.16

Patient ID	Coverage (bp)	On target (%)	Mean depth (x)	Uniformity (%)
EC-BS91	94165	67.6	1528	97.33
EC-BS92	236347	74.45	4555	97.26
EC-BS93	624301	67.14	10625	96.19
EC-BS95	150683	89.06	3410	96.16

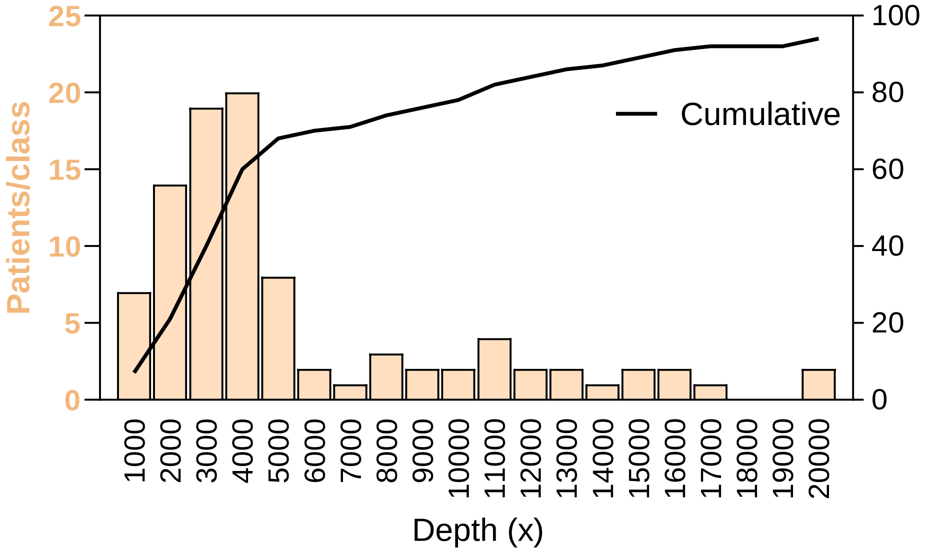

Figure S1. Distribution of sequencing depth in 1000x-wide classes (orange bars) and cumulative sum (black line) according to the number of patients belonging to each class.

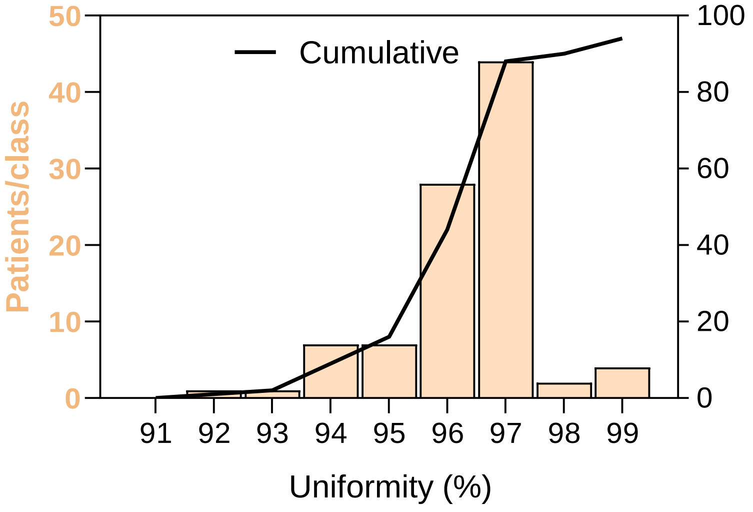

Figure S2. Distribution of sequencing uniformity in 1%-wide classes (orange bars) and cumulative sum (black line) according to the number of patients belonging to each class.

Table S3. Pathogenic variants in the POLE exonuclease domain.

Patient's code	POLE exon	Nucleotide substitution	Protein change	Allele Frequency
EC-BS20	9	c.857C>G	P286R	39%
EC-BS82	9	c.857C>G	P286R	17%
EC-BS83	9	c.857C>G	P286R	34%
EC-BS84	9	c.857C>G	P286R	21%
EC-BS5	9	c.890C>T	S297F	15%
EC-BS70	9	c.890C>T	S297F	22%
EC-BS72	13	c.1231G>C	V411L	31%
EC-BS29	13	c.1231G>T	V411L	38%
EC-BS48	13	c.1231G>T	V411L	42%
EC-BS18	13	c.1231G>T	V411L	31%
EC-BS85	13	c.1231G>T	V411L	34%
EC-BS91	14	c.1366G>C	A456P	30%
EC-BS93	14	c.1366G>C	A456P	40%
EC-BS86	14	c.1366G>C	A456P	34%
EC-BS92	14	c.1376C>T	S459F	39%
EC-BS48	14	c.1376C>T	S459F	44%

Table S4. Non-pathogenic variants in POLE exons 9-14, 19, 20, 26, 30, 36, 37, 39, 42, and 46.

Patient's code	POLE exon	Nucleotide substitution	Protein change	Allele Frequency
EC-BS70	12	c.1149G>A	M383I	11%
EC-BS64	12	c.1187A>G	E396G	28%
EC-BS1	14	C.1394C>T	A465V	36%
EC-BS 14	26	c.3271G>A	E1091K	5%
EC-BS 84	30	c.3703C>A	L1235I	24%
EC-BS 87	30	c.3595G>A	E1199K	7%
EC-BS 2	36	c.4576C>A	L1526I	48%
EC-BS 18	36	c.4666C>T	R1536W	19%
EC-BS 34	37	c.4798A>G	I1600V	9%
EC-BS 87	37	c.4735C>T	R1579C	12%
EC-BS 87	37	c.4876C>T	R1626C	7%
EC-BS 94	37	c.4849A>G	I1617V	9%
EC-BS 9	42	c.5794C>T	R1932C	6%
EC-BS 82	42	c.5705C>A	S1902T	11%
EC-BS 18	46	c.6418G>A	E2140K	50%
EC-BS 52	46	c.6418G>A	E2140K	51%
EC-BS 59	46	c.6418G>A	E2140K	51%
EC-BS 72	46	c.6439C>T	P2147S	30%
EC-BS 82	46	c.6380G>A	R2127Q	16%
EC-BS 91	46	c.6494G>A	R2165H	45%

Table S5. Clinicopathological characteristics of “multiple-classifier” EC patients of the Brescia cohort.

	**Patient's code**					
	EC-BS5	EC-BS29	EC-BS70	EC-BS72	EC-BS85	EC-BS1
**Molecular subtype**	POLE-mut	POLE-mut	POLE-mut	POLE-mut	POLE-mut	MMR-D
**Age (years)**	70	64	31	44	63	47
**Histotype**	end	non-end	non-end	end	end	end
**Myometrial invasion**	M2	M1	M1	M1	M2	M2
**Tumor Grade**	G3	G3	G3	G2	G3	G3
**LVSI**	yes	no	yes	yes	yes	yes
**Lymph node metastasis**	pos	neg	neg	neg	neg	pos
**FIGO stage**	IIIC	IB	IA	IA	IB	IV
**Risk Group (ESGO/ESTRO/ESP 2020)***	High	High	High	High-int.	High-int.	High
**POLE**	**mut**	**mut**	**mut**	**mut**	**mut**	wt
**MMR**	**deficient**	proficient	**deficient**	**deficient**	**deficient**	**deficient**
**p53**	wt	**abn**	wt	wt	wt	**abn**
**CTNNB1**	wt	wt	wt	wt	wt	wt
**ARID1A**	loss	loss	loss	present	present	loss
**L1CAM**	neg	pos	neg	neg	neg	neg
**ER (%)**	40	0	90	0	70	0
**PR (%)**	15	0	70	5	30	0
**KI-67 (%)**	50	65	30	15	60	45
**Degree of inflammation**	prominent	moderate	prominent	moderate	prominent	weak
**Recurrence**	no	no	yes	no	no	no
**Adjuvant Therapy**	CT + RT	CT	CT	RT	RT	CT
**Time to recurrence after CT (months)**	**-**	-	42	-	-	-
**Status at the last follow up**	NED	NED	NED	NED	NED	NED
**DSS (months)**	78	145	78	89	165	168

RT, radiotherapy; CT, platinum-based adjuvant chemotherapy; NED, no evidence of disease.

*Concin N. et al. [1]

Table S6. List of variants in the CTNNB1 exon 3.

Patient's code	Nucleotide substitution	Protein change	Allele Frequency	Coverage	CTNNB1 ClinVar classification	CTNNB1 variants already described in EC
EC-BS3	c.97T>C	S33P	11%	14183	Likely path.	Kim G et al. [2]
EC-BS61	c.97T>G	S33A	39%	3671	Likely path.	Kim G et al. [2]
EC-BS27	c.98C>A	S33Y	22%	1073	Path./Likely path.	Kim G et al. [2]
EC-BS34	c.98C>A	S33Y	11%	5229	Path./Likely path.	Kim G et al. [2]
EC-BS73	c.98C>A	S33Y	6%	2150	Path./Likely path.	no
EC-BS37	c.98C>T	S33F	24%	2030	Path./Likely path., other	Kim G et al. [2]
EC-BS3	c.100G>A	G34R	5%	14190	Likely path.	Liu Y et al. [3]
EC-BS53	c.198G>A	W66	23%	15842	Path.	no
EC-BS79	c.100C>G	S37C	31%	2936	Path./Likely path.	Kim G et al. [2]
EC-BS69	c.101G>T	G34V	33%	293	Conflicting interpretations of pathogenicity	Kim G et al. [2]
EC-BS17	c.110 C>T	S37F	20%	7384	Path./Likely path.	Liu Y et al. [3]
EC-BS40	c.121A>G	T41A	24%	2482	Conflicting interpretations of pathogenicity, other	Kim G et al. [2]
EC-BS46	c.122C>T	T41I	7%	4719	Path./Likely path.	Kim G et al. [2]
EC-BS53	c.134C>A	S45Y	9%	9566	Path./Likely path.	no

Path, pathogenic.

Table S7. Univariable survival analysis for disease-specific survival (DSS) and progression-free survival (PFS) according to additional biomarkers within each molecular subtype of the Brescia cohort.

Variables	MMR-D (n. 34)			NSMP (n. 20)			p53abn (n. 25)		
	HR	95%CI	p-value	HR	95%CI	p-value	HR	95%CI	p-value
**DSS**									
L1CAM (pos vs neg)	1.23	0.15-10.0	0.848	3.93	1.03-14.9	**0.045**	1.54	0.50-4.75	0.456
ARID1A (loss vs present)	0.64	0.15-2.70	0.500	0.37	0.10-1.32	0.127	0.63	0.08-4.85	0.659
CTNNB1 (mut vs wt)	1.68	0.34-8.35	0.529	0.52	0.14-1.98	0.334	-	-	-
ER (10% of increment)	0.93	0.77-1.12	0.423	0.83	0.66-1.05	0.118	0.99	0.80-1.23	0.952
PR (10% of increment)	0.94	0.76-1.16	0.569	0.77	0.60-1.00	0.052	0.60	0.29-1.24	0.169
Ki67 (10% of increment)	0.72	0.48-1.07	0.107	1.2	0.93-1.55	0.159	1.04	0.86-1.26	0.707
Inflammation (m/p vs a/w)	0.33	0.07-1.65	0.176	0.76	0.31-1.89	0.557	0.82	0.31-2.17	0.686
**PFS**									
L1CAM (pos vs neg)	0.62	0.08-4.74	0.616	4.12	1.22-13.9	**0.023**	1.31	0.47-3.66	0.605
ARID1A (loss vs present)	0.68	0.23-2.04	0.548	0.70	0.25-1.97	0.498	0.59	0.08-4.48	0.609
CTNNB1 (mut vs wt)	2.03	0.61-6.22	0.263	1.21	0.43-3.39	0.718	-	-	-
ER (10% of increment)	1.03	0.89-1.20	0.723	0.88	0.75-1.03	0.110	1.01	0.82-1.24	0.931
PR (10% of increment)	1.05	0.90-1.22	0.611	0.84	0.70-1.00	**0.045**	0.59	0.30-1.19	0.140
Ki67 (10% of increment)	0.82	0.61-1.10	0.240	1.13	0.91-1.38	0.265	1.02	0.84-1.25	0.816
Inflammation (m/p vs a/w)	0.28	0.08-1.02	0.054	0.68	0.22-2.17	0.520	0.72	0.28-1.85	0.499

The POLE group was not analysed due to the almost total absence of events (none for DSS and one for PFS).
Inflammation: m/p, moderate/prominent; a/w, absent/weak.
HR, hazard ratio; CI, confidence interval. Significant p-values are in bold.

References

1. Concin N, Matias-Guiu X, Vergote I, Cibula D, Mirza MR, Marnitz S, Ledermann J, Bosse T, Chargari C, Fagotti A, Fotopoulou C, Gonzalez Martin A, Lax S, Lorusso D, Marth C, Morice P, Nout RA, O'Donnell D, Querleu D, Raspollini MR, Sehouli J, Sturdza A, Taylor A, Westermann A, Wimberger P, Colombo N, Planchamp F, Creutzberg CL. ESGO/ESTRO/ESP guidelines for the management of patients with endometrial carcinoma. *Int J Gynecol Cancer* **2021**, 31, 12-39. doi:10.1136/ijgc-2020-002230.

2. Kim G, Kurnit KC, Djordjevic B, Singh C, Munsell MF, Wang WL, Lazar AJ, Zhang W, Broaddus R. Nuclear β-catenin localization and mutation of the CTNNB1 gene: a context-dependent association. *Mod Pathol*. **2018**, 31, 1553-1559. doi: 10.1038/s41379-018-0080-0.
3. Liu Y, Patel L, Mills GB, Lu KH, Sood AK, Ding L, Kucherlapati R, Mardis ER, Levine DA, Shmulevich I, Broaddus RR, Zhang W. Clinical significance of CTNNB1 mutation and Wnt pathway activation in endometrioid endometrial carcinoma. *J Natl Cancer* Inst. **2014**, 106, dju245. doi: 10.1093/jnci/dju245.
